# Effect of Mask Geometry Variation on Plasma Etching Profiles †

**DOI:** 10.3390/mi14030665

**Published:** 2023-03-16

**Authors:** Josip Bobinac, Tobias Reiter, Julius Piso, Xaver Klemenschits, Oskar Baumgartner, Zlatan Stanojevic, Georg Strof, Markus Karner, Lado Filipovic

**Affiliations:** 1 Christian Doppler Laboratory for Multi-Scale Process Modeling of Semiconductor Devices and Sensors at the Institute for Microelectronics, TU Wien, Gußhausstraße 27-29/E360, 1040 Vienna, Austria; bobinac@iue.tuwien.ac.at (J.B.); reiter@iue.tuwien.ac.at (T.R.); 2 Institute for Microelectronics, TU Wien, Gußhausstraße 27-29/E360, 1040 Vienna, Austria; piso@iue.tuwien.ac.at (J.P.); klemenschits@iue.tuwien.ac.at (X.K.); 3 Global TCAD Solutions GmbH, Bösendorferstraße 1, Stiege 1, Top12, 1010 Vienna, Austria; o.baumgartner@globaltcad.com (O.B.); z.stanojevic@globaltcad.com (Z.S.); g.strof@globaltcad.com (G.S.); m.karner@globaltcad.com (M.K.)

**Keywords:** SF_6_/O_2_, plasma etching, high-aspect-ratio structures, 3D integration, mask tapering, mask faceting, mask geometry, modeling and simulation, process TCAD

## Abstract

It is becoming quite evident that, when it comes to the further scaling of advanced node transistors, increasing the flash memory storage capacity, and enabling the on-chip integration of multiple functionalities, “there’s plenty of room at the top”. The fabrication of vertical, three-dimensional features as enablers of these advanced technologies in semiconductor devices is commonly achieved using plasma etching. Of the available plasma chemistries, SF_6_/O_2_ is one of the most frequently applied. Therefore, having a predictive model for this process is indispensable in the design cycle of semiconductor devices. In this work, we implement a physical SF_6_/O_2_ plasma etching model which is based on Langmuir adsorption and is calibrated and validated to published equipment parameters. The model is implemented in a broadly applicable in-house process simulator ViennaPS, which includes Monte Carlo ray tracing and a level set-based surface description. We then use the model to study the impact of the mask geometry on the feature profile, when etching through circular and rectangular mask openings. The resulting dimensions of a cylindrical hole or trench can vary greatly due to variations in mask properties, such as its etch rate, taper angle, faceting, and thickness. The peak depth for both the etched cylindrical hole and trench occurs when the mask is tapered at about 0.5°, and this peak shifts towards higher angles in the case of high passivation effects during the etch. The minimum bowing occurs at the peak depth, and it increases with an increasing taper angle. For thin-mask faceting, it is observed that the maximum depth increases with an increasing taper angle, without a significant variation between thin masks. Bowing is observed to be at a maximum when the mask taper angle is between 15° and 20°. Finally, the mask etch rate variation, describing the etching of different mask materials, shows that, when a significant portion of the mask is etched away, there is a notable increase in vertical etching and a decrease in bowing. Ultimately, the implemented model and framework are useful for providing a guideline for mask design rules.

## 1. Introduction

As transistor scaling of planar field-effect transistors (FETs) started to reach its physical limit, vertical gate geometries were developed to overcome them and the FinFET structure was born, which dominates the current landscape at advanced technology nodes [1,2]. IBM has also recently announced that they are working on a vertical transistor architecture, in an attempt to effectively continue scaling along Moore’s Law [3] by taking advantage of the vertical dimension. The microelectronics industry has also been looking for solutions for the integration of applications beyond memory and logic on the same die, such as radio frequency (RF) circuits, power electronics, and sensors. This type of three-dimensional (3D) integration, termed More-than-Moore [4], requires functional diversification and electrical connections through the entire thickness of a wafer, which can be several hundred micrometers, typically using through-silicon vias (TSVs) [5,6,7]. Furthermore, the memory capacity of modern 3D NAND-based storage is limited by the total number of sequential conductive and insulating stacked layers which form the NAND gate. The fabrication of these gates relies on etching through silicon dioxide (SiO_2_) and silicon nitride (Si_3_N_4_) stacks [8]. Therefore, to increase the memory capacity, more stacks need to be used, which is currently limited to 128 [9]. This limitation is primarily a consequence of the etching step, which still relies on a plasma chemistry. Finally, plasma-etched nanowire arrays are emerging as a new class of high-aspect-ratio microstructures in silicon for next-generation energy harvesting such as thermoelectrics [10] and for storage devices, such as lithium ion batteries [11,12].

It is becoming evident that further scaling of digital logic, increase in memory storage capacity, improvements in energy harvesting and battery technology, and the functional integration on a single die all rely on vertical integration. In 1959, Richard Feynman famously observed that “there’s plenty of room at the bottom” to indicate the tremendous importance of scaling and the revolutionary potential of exploring and exploiting quantum phenomena in semiconductor devices [13]. However, for additive manufacturing (AM), it is becoming quite evident that the opposite is simultaneously true and that vertical integration is principally important, meaning that “there’s plenty of room at the top” [14,15,16].

To fulfill the promise of efficiently using the vertical dimension, the fabrication of ever-higher high-aspect-ratio (HAR) structures is becoming critical. At the same time, the demand for HAR geometries is increasing and testing every possible setting experimentally is incredibly expensive and time-consuming, which is why physical models have become an indispensable tool in the design cycle. Plasma etching is one of the most frequently used fabrication steps to generate such structures; however, the process is quite complex and providing a predictive model which includes all relevant physics is a challenging task. Nevertheless, physical models are essential since their only alternative involves repeated experiments which involve many trial-and-error procedures, due to the sheer complexity of plasma etching [17].

The increased need for etching of HAR structures is driving further mask innovation [18]. There are many nonidealities which arise due to variations in mask geometry, such as mask erosion and undercutting during etching. Additionally, variations in lithography, which is becoming more complex, can cause significant mask variations. Physical simulations are essential in order to be able to qualify and quantify the impact of these variations on the final fabricated profile. The model used for analyzing the impact also must be integrated within a complete process simulator and combined with many other processing steps in order to generate entire devices and even circuits. Only this way, can a realizable design-technology co-optimization (DTCO) strategy be applied to study future device designs. This paper, for the first time, uses a physical model which includes particle reflections and is implemented in a fully fledged process simulation framework, to study the impact of several mask variations in the commonly applied SF_6_/O_2_ plasma etching system. Most previous studies make several assumptions about the mask [17], but this is becoming insufficient to fully identify its impact on the final etched features. Our approach shows that the SF_6_/O_2_ model and framework implementation can capture the complete picture of the simulated material stack and system.

In order to fabricate HAR structures, deep reactive ion etching (DRIE) is commonly applied, which consists of cycles of plasma etching followed by a conformal deposition of a passivating layer [19]. The passivating layer at the bottom of the structure is sputtered away by ion impact, while the sidewalls remain covered and prevent lateral etching. The implemented model represents a commonly applied plasma chemistry which involves using SF_6_ in a plasma, whereby fluoride (F) is the dominant chemical etch species [20,21]. The addition of O_2_ forms a protective layer during the etch phase. The protective layer is inert to chemical reactions with the fluoride and inhibits etching. The ionic species, which are formed in the plasma, are accelerated vertically, resulting in a highly directional sputter etching of the substrate. The ions also remove the oxidized surface at the bottom of the trench, allowing for the fluoride to etch at these locations chemically. Overall, analogous to the impact of the deposited conformal layer, the result is that the sidewalls are shielded, as ions do not tend to sputter the sidewall, since they are primarily directed towards the bottom, allowing to obtain HAR holes. The precise geometry of the etched hole depends on the mask opening and its shape. In DRIE, since a passivating layer is directly deposited on the sidewall, it is not sputtered by ion impact and the entire sidewall effectively serves as a mask in the subsequent etching step. Therefore, understanding the impact of this mask (i.e., all layers above the layer to be etched) on the etch rate is crucial for controlling and optimizing the process.

In order to assess the impact of the mask layer on the final etched profile of an HAR hole, we implemented the physical SF_6_/O_2_ plasma etching model in the level set-based process simulator ViennaPS [22], which uses the level set library ViennaLS [23] for surface storage and to calculate the level set equation, as well as the ray tracing library ViennaRay [24] for Monte Carlo ray tracing. The model was calibrated and validated by matching the simulated profiles of cylindrical holes to experimental measurements from literature [25]. The model was then applied to qualitatively study the influence of a tapered mask and mask faceting, mask etch rate, and mask thickness on the etched hole profiles.

## 2. Simulation Workflow

**Figure 1:**
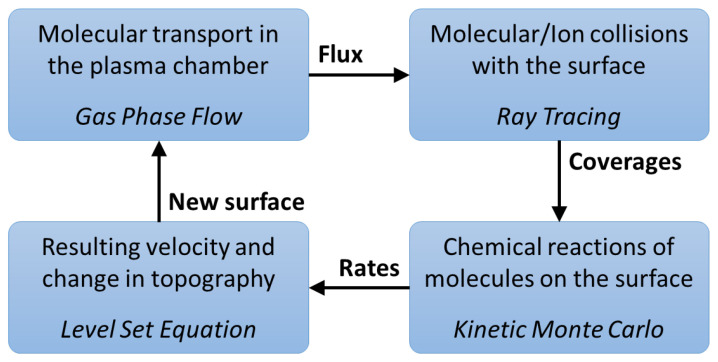
Schematic representation of the employed simulation flow. The starting point is obtaining fluxes which capture particle transport in the plasma chamber. The fluxes are then used to obtain the particle fluxes which impinge on each surface element and to calculate their coverage, which in turn serves as an input for modeling reactions on the surface. Reactions at the surface produce rates which are used to obtain velocities and move (advect) the surface by solving the level set equation.

**Figure 2:**
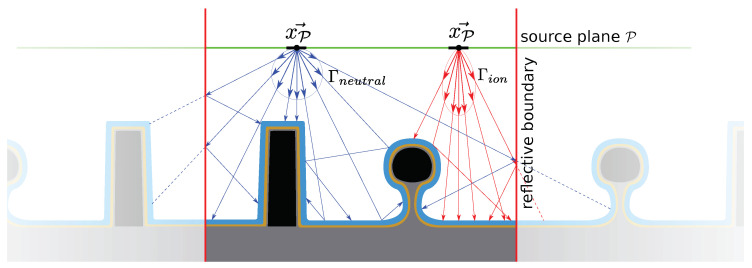
Schematic depiction of particle rays being traced from the source plane to the surface. The particles describe either neutral atoms or molecules (blue) or ions (red), governed by the source distribution fluxes $\Gamma_{n}$ and $\Gamma_{i}$, respectively. The fluxes define the distribution of particle directions and energies. Specular reflections are shown for ions, and diffuse reflections for neutral species.

The simulation of plasma etching requires the definition of multiple materials and their interfaces, which we apply using the level set approach, as shown in the workflow given in Figure 1. The atoms, molecules, and ions are stochastically modeled using particles which represent several hundred or thousand physical atoms, molecules, or ions. Their movement in the plasma reactor is treated using continuum transport equations [26], while their motion near the surface is treated ballistically using ray tracing, as continuum equations are not valid at that scale [26,27]. Once a particle strikes the surface, represented using a level set description, a kinetic surface chemistry model is applied at the intersection of a particle ray and the surface, which includes sputtering, chemical etching, and reflections. This flow follows the typical top-down Monte Carlo ray tracing depicted in Figure 2 and described in [27,28,29,30]. Alternatively, bottom-up simulations have also been used for these types of simulations, albeit with severe restrictions on modeling particle reflections [17].

### 2.1. Implicit Surface Representation

The level set method is a form of implicit surface representation, where the surface is described by a signed distance function (SDF) $\phi (\vec{x})$ defined at all points in space [31,32]. The function is typically defined on a Cartesian grid with signed distance values defined at each point. The surface is then located where the SDF is equal to a specific scalar value, typically zero. In this case, the surface is said to be the zero level set. The SDF $\phi (\vec{x})$ is constructed based on the signed distance *d* of a domain point $\vec{x}$ from the surface *S* bounding the volume *M*:

(1) $$\phi (\vec{x})=\left\{\begin{array}{cc} -d, & \vec{x}\in M\\ 0, & \vec{x}\in S\\ d, & \vec{x}\notin M \end{array}\right.$$

The sign of $\phi (\vec{x})$, whether negative or positive, indicates whether the point is inside or outside the volume *M*, respectively. In this way, it is immediately known whether a point is inside or outside the volume, simply by examining the sign of the SDF. The time evolution of the surface is described by the surface normal velocity $v(\vec{x})$. For a typical geometry with nonconstant SDF gradients, the gradient is used to normalize $v(\vec{x})$, which leads to the level set equation:

(2) $$\frac{\delta \phi (\vec{x},t)}{\delta t}+v(\vec{x})\left|\nabla \phi (\vec{x},t)\right|=0$$

Since Equation (2) is a form of the Hamilton–Jacobi equations, many algorithms exist which are able to solve it using finite-difference schemes [33,34]. The resulting velocity is subsequently used to update the SDF $\phi (\vec{x})$ and these values are typically stored at the points defined on a regular grid. As the surface evolves, the points remain at the same position while their SDF value changes [29].

### 2.2. Surface Kinetics Model

To describe the temporal simulation of feature-scale SF_6_/O_2_ plasma etching, we consider the rates of ions and neutral particles-oxygen and fluorine-along with their surface coverages at each time step. Ray tracing is employed to compute particle impingement rates which are, in turn, used to compute the surface coverage of relevant species and ultimately to calculate the surface velocity or etch rate (ER). The etch rate (ER) is then used to extract the effective velocities at all defined points $\vec{x}$ in space $v(\vec{x})$, which are used to solve the level set Equation (2). The ER is determined by three physical phenomena:

- Chemical etching: The fluorine reacts with the exposed silicon surface, releasing SiF_4_ as a by-product [35]. The chemical reaction which governs this process above 100 °C is given by [36]
  (3) $$\mathrm{Si}+2{\mathrm{SF}}_{6(g)}⭢{\mathrm{SiF}}_{4(g)}+2{\mathrm{SF}}_{4(g)}.$$
- Physical sputtering: Ions in the chamber will strike the wafer surface, resulting in the physical sputtering of silicon atoms away from the surface. This will, however, only take place if the incident ions have an energy above a critical threshold energy $E_{th}$.
- Ion-enhanced etching, also known as reactive ion etching (RIE): fluorine-saturated silicon surfaces are more prone to being physically sputtered, meaning that the critical threshold energy to emit a silicon atom is reduced, when compared to the physical sputtering process [37].

Therefore, the model must be able to track the coverages of fluorine and oxygen on the silicon surface, given by $\theta_{F}$ and $\theta_{O}$, respectively. These are calculated using a Langmuir–Hinshelwood type surface-site balance equation [38] which is given by:

(4) $$\sigma_{Si}\frac{d\theta_{F}}{dt}=\gamma_{F}\Gamma_{F}\left(1-\theta_{F}-\theta_{O}\right)-k\sigma_{Si}\theta_{F}-2Y_{ie}\Gamma_{i}\theta_{F}$$

(5) $$\sigma_{Si}\frac{d\theta_{O}}{dt}=\gamma_{O}\Gamma_{O}\left(1-\theta_{F}-\theta_{O}\right)-\beta \sigma_{Si}\theta_{O}-Y_{O}\Gamma_{i}\theta_{O}$$

Here, $\sigma_{Si}$ is the surface site density of silicon; $\Gamma_{F}$, $\Gamma_{O}$, and $\Gamma_{i}$ are the fluorine, oxygen, and ion fluxes, respectively; $\gamma_{F}$ and $\gamma_{O}$ are the sticking coefficients for fluorine and oxygen on a clean silicon surface, respectively; *k* is the chemical etch reaction rate constant; β is the oxygen recombination rate constant; and $Y_{ie}$ and $Y_{O}$ are the total ion-enhanced and oxygen etching rates, respectively, which are linked to the ion energies in the reactor [27].

Assuming steady-state conditions, Equations (4) and (5) can be set to zero, resulting in the following equations for the surface coverage values:

(6) $$\theta_{F}={\left[1+\left(\frac{k\sigma_{Si}+2Y_{ie}\Gamma_{i}}{\gamma_{F}\Gamma_{F}}\right)\left(1+\frac{\gamma_{O}\Gamma_{O}}{\beta \sigma_{Si}+Y_{O}\Gamma_{i}}\right)\right]}^{-1}$$

(7) $$\theta_{O}={\left[1+\left(\frac{\beta \sigma_{Si}+Y_{ie}\Gamma_{i}}{\gamma_{O}\Gamma_{O}}\right)\left(1+\frac{\gamma_{F}\Gamma_{F}}{k\sigma_{Si}+2Y_{ie}\Gamma_{i}}\right)\right]}^{-1}$$

The steady-state assumption is valid since the incoming fluxes of all particles are relatively high, on the order of 10${}^{16}$–10${}^{19}$ cm^−2^s^−1^ and the velocities of particles in the plasma are much faster than the ER, which is on the order of several nanometers per second [17]. Since it is well established that oxygen does not directly cause the removal of silicon, meaning it does not contribute to the etch rate, only the fluorine and ion species result in surface removal. Numerically, this means that the oxygen species only serves to reduce the concentration of fluorine from the surface, as can be seen from the right side of Equation (6), where increasing oxygen flux $\Gamma_{O}$ causes a reduction in $\theta_{F}$. Finally, the etch rate is given by

(8) $$\mathrm{ER}=\frac{1}{\rho_{Si}}\left(\frac{k\sigma_{Si}\theta_{F}}{4}+Y_{p}\Gamma_{i}+Y_{ie}\Gamma_{i}\theta_{F}\right),$$

where $\rho_{Si}$ is the silicon density. The first, second, and third terms in the brackets of Equation (8) reflect the chemical etching, physical sputtering, and ion-enhanced etch components, respectively, as described above.

## 3. Model Calibration

The implemented model was calibrated to the experimental data from Belen et al. [25] to ensure that the model could correctly capture the changes in the chamber gas composition, the applied RF bias, the chamber pressure, and the feature size. The fluorine and oxygen fluxes, $\Gamma_{F}$ and $\Gamma_{O}$, respectively, were varied to model the composition of the chamber gas. The ion energy encapsulated the effect of the applied RF bias, and the pressure was not modeled directly but was instead included in the model by matching the simulations to experimentally obtained profiles. The calibration procedure for the model is described in more detail in Belen et al. [25] and Bobinac et al. [39]. This calibration was performed on a cylindrical mask with top and bottom diameters of 0.4 μm and 0.35 μm, respectively, and a thickness of 1.2 μm.

**Figure 3:**
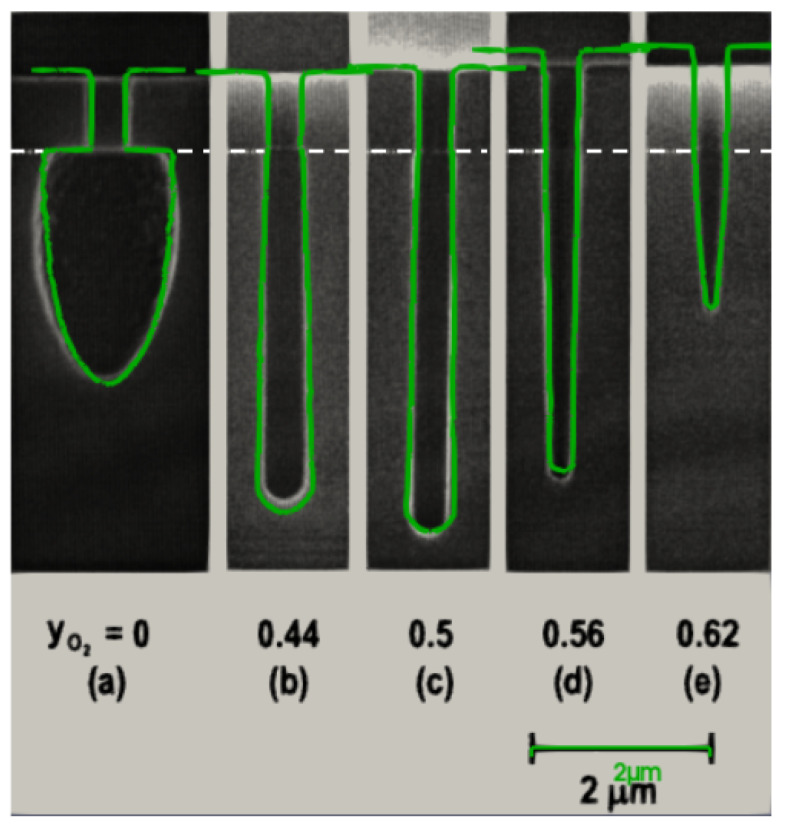
Diagonal slices through a 3D-simulated (green) and SEM cross sections of cylindrical hole profiles from Belen et al. [25] (black). The increasing presence of O_2_ hinders lateral etching while initially enhancing vertical etching (**a**–**c**). Further increasing the O_2_ flux leads to negative sidewall tapering and a reduction in the vertical etching (**d**,**e**). The oxide mask–Si interface is depicted with a white dashed line. (Reprinted with permission from [25]). Copyright 2005, American Vacuum Society.

In Figure 3a, we show the capability of the model to capture more isotropic etch profiles, where no oxygen is present in the feed gas. The model is able to capture complex phenomena, such as a temporary increase in the vertical etch rate, when the ratio of O_2_ gas to SF_6_ gas increases from 0.44 to 0.5. Further increasing the oxygen content reduces the overall etch rate, as intuitively expected. Figure 3 shows the scanning electron microscopy (SEM) cross sections from Belen et al. [25] overlaid with the calibrated model in green. From the setups containing oxygen in the feed gas, (Figure 3b–e), the lowest oxygen content results in the highest lateral etch rate, shown in Figure 3b. When the oxygen flux is increased, the sidewall passivation becomes more pronounced and lateral etching is decreased. Increased sidewall oxygen coverage directs more fluorine flux towards the bottom of the hole, resulting in a higher vertical etch yield, shown in Figure 3c. Further increasing the oxygen content results in a negative (inward) sidewall tapering and an inhibition of vertical etching, as observed in Figure 3d and, to a greater extent, in Figure 3e.

**Figure 4:**
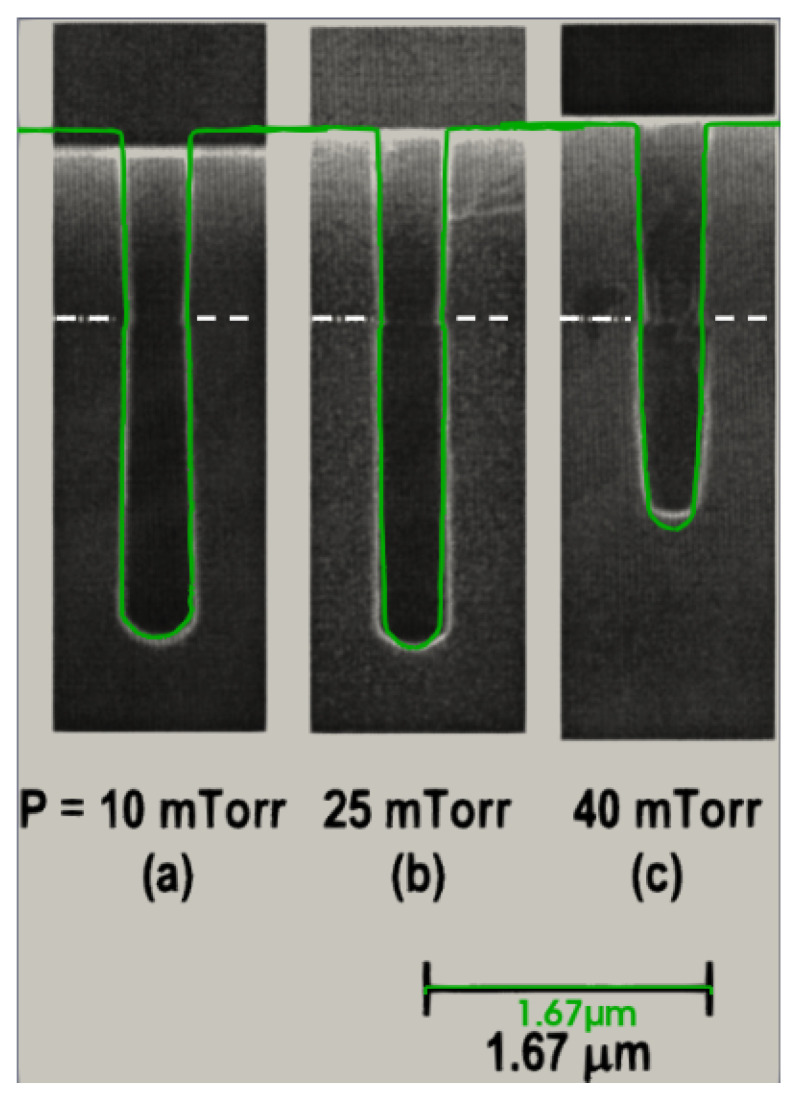
Diagonal slices through a 3D-simulated (green) and experimental (black) cylindrical hole profiles at increasing chamber pressures: (**a**) 10 mTorr; (**b**) 25 mTorr; (**c**) 40 mTorr. The oxide mask–Si interface is depicted with a white dashed line. (Reprinted with permission from [25]). Copyright 2005, American Vacuum Society.

In order to capture the changing chamber pressure in the model, it must be understood that increasing the pressure results in a direct increase in the fluorine and oxygen fluxes (neutral species) and a reduction in the ion flux. At low pressures, the inhibiting effect of oxygen coverage is less significant and we observe outward sidewall tapering, as depicted in Figure 4. As the pressure increases, despite a decreased ion flux, vertical etching slightly increases due to the same effect of more fluorine reflecting towards the bottom, depicted in Figure 4b. Further increasing the pressure inhibits both vertical and lateral etching and changes the sidewall tapering from negative (outward) to positive (inward), as depicted in Figure 4c.

## 4. Results and Discussion

**Figure 5:**
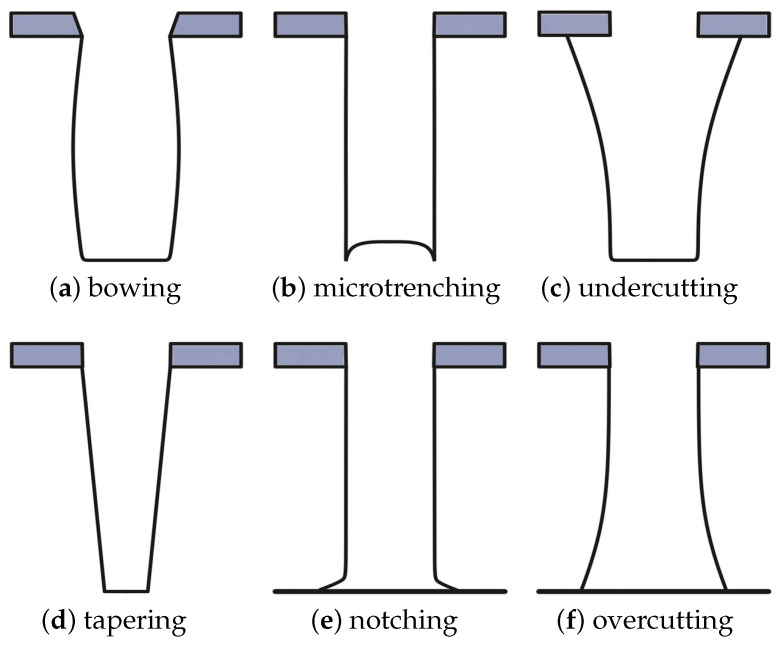
Effects of plasma etching on the final profile: (**a**) bowing due to mask faceting; (**b**) microtrenching due to an enhanced ion flux along the sidewall; (**c**) undercutting due to an isotropic component in the etch process; (**d**) sidewall tapering due to sidewall inhibition or deposition of polymer on the sidewall; (**e**) notching at the interface with an etch-stop layer due to inadequate sidewall passivation or charging effects; (**f**) Re-entrant profile (overcutting) due to inadequate sidewall passivation and/or ion scattering. (Reprinted with permission from Donnelly and Kornblit [38]).

There are many reasons why masks may not have the perfect geometry, as is often assumed in plasma simulations [40]. The physical model presented here, using top-down Monte Carlo ray tracing, is able to qualitatively reproduce and predict many phenomena observed in plasma etched profiles. This includes loading, RIE lag, mask faceting, microtrenching, undercutting, sidewall tapering, notching, and overcutting, depicted in Figure 5 from Donnelly and Kornblit [38]. To quantify these effects, typically some calibration is necessary in order to ensure that the model can accurately represent the specific equipment being used.

We finally use our model for a qualitative insight into the impact of varying mask geometries on the etched feature profile. The parameters from Table 1 were applied to the model, which was calibrated to physical conditions of a total gas flow rate in the chamber of 80 sccm at a pressure of 25 mTorr, an inductive coil power of 800 W, and an RF-bias voltage of −120 V at a wafer temperature of 5 °C. The gas flow rate of O_2_ was varied from 35 sccm to 50 sccm, corresponding to the oxygen yield y_O_2__ varying from 0.44 to 0.62, respectively, with the rest of the flow being that of SF_6_ as described in [25].

### 4.1. Impact of Mask Tapering

**Figure 6:**
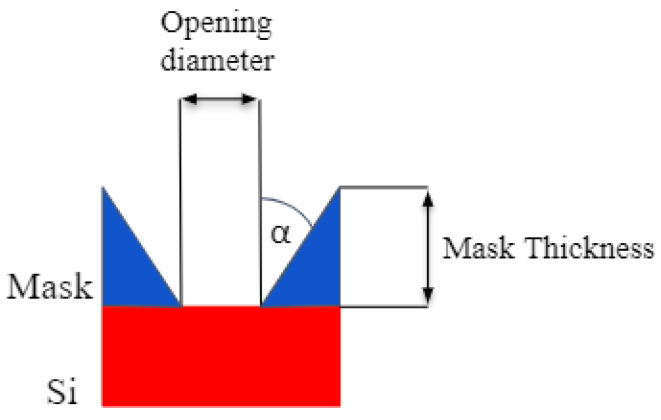
Illustration of the taper angle α. For the impact of the mask tapering angle, small angles on relatively thick masks are considered. In mask faceting, ion bombardment sputters away the top mask corner and results in an effective taper angle as well. However, the angles tend to be larger and the impact is analyzed for thin masks.

**Figure 7:**
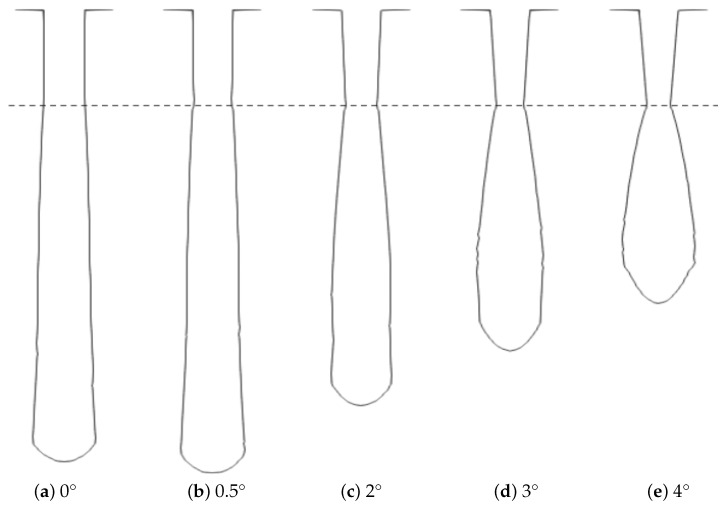
Cross sections of the etched cylindrical holes obtained by varying the mask tapering from perfectly vertical at 0° to 4° for y_O_2__ = 0.5. Both the increased vertical etch rate with slight tapering and the effects of bowing are reproduced.

Using the model parameters listed in Table 1, we studied the impact of tapering of a 1.2 μm thick cylindrical mask with an opening diameter of 0.4 μm. The mask tapering angle, illustrated in Figure 6, was varied from 0° to 4° during the simulations in order to assess its impact on the etched profile, with the results shown in Figure 7. A similar study was performed on the impact of fluorocarbon etching of SiO_2_ and halogen gas etching of Si in 3D NAND memory stacks [41,42]. These studies were able to qualify the effects of the previously etched layers on the etching rate and sidewall tapering of the bottom of the HAR structure. The predictive modeling of these phenomena helps to optimize the process by allowing for a slight outward tapering in the SiO_2_ layer, followed by a slight inward tapering of the silicon layer.

The initial increase in the mask taper angle from 0° to 0.5° enhanced the vertical etching, since more ions were directly reflected towards the bottom. Effectively, this was a similar phenomenon as the one leading to microtrenching, since it involved ions grazing off of the sidewalls and contributing to a faster etch rate at the hole bottom. As the taper angle was increased, the ions redirected off of the mask at a more lateral angle, resulting in an increased sidewall etching and bowing. As the mask taper angle was further increased, the ions were redirected towards the sidewall closer to the top of the hole, shifting the bowing effect closer to the mask layer.

**Figure 8:**
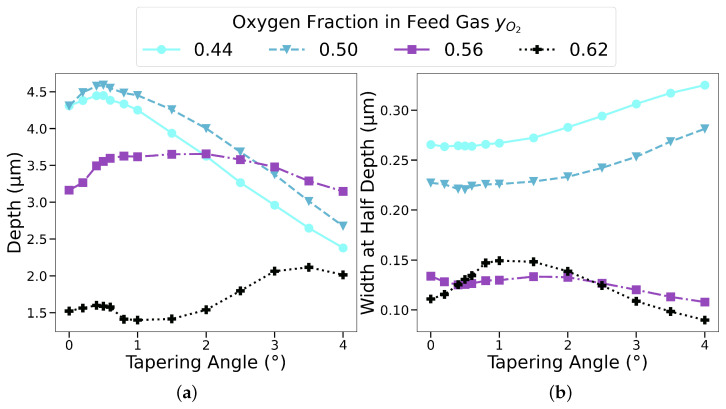
(**a**) Depth and (**b**) width at half-depth (WAHD) of a cylindrical hole as the mask tapering angle was varied from 0° to 4°. The depth is maximized when the WAHD reaches its minimum for all the gas compositions. For lower-oxygen-content settings, where the passivation does not attenuate the changes in tapering, the depth peaks after an initial angle increase and decreases significantly as the angle increases further, while the WAHD reaches a minimum after an initial angle increase and increases significantly with a further angle increase. Since the most significant changes were expected for small taper angles, we used more sample points in the range from 0° to 1°.

In Figure 8, we note the effective changes in the final cylindrical trench geometry for varied chamber gas compositions detailed in Table 1, represented as the final depth and the width at half-depth (WAHD), while the mask taper angle was varied. The maximum depth for y_O_2__ = 0.44 and y_O_2__ = 0.5 was reached when the tapering angle was low but not zero, at around 0.5°. A further increase in the oxygen concentration in the feed gas shifted the maximum depth towards higher angles. The bowing effect, captured by the WAHD, was more pronounced in the low-oxygen-content settings due to a weaker passivation effect on the sidewalls. The WAHD was shown to be at its minimum for the mask taper angles at which the hole depths were at their maximum. This was shown to be the case for all chamber gas compositions as these were the points where the most ions hit the very bottom of the hole with high energy. Therefore, we can conclude that this is a physical phenomenon which strongly depends on the mask geometry. Apart from the y_O_2__ = 0.62, which exhibited a different behavior due to a significantly reduced vertical etching, reducing the tapering angle below 1° resulted in more ions being reflected towards the bottom, while increasing it resulted in a bowing closer to the mask layer and away from the middle of the hole depth.

### 4.2. Impact of Mask Faceting

In the previous section, we studied the impact of mask tapering, or the effect of having a layer on top of the etched layer which had a tapered sidewall profile. However, even if the mask initially has no tapering at the start of the plasma etching step, some faceting at the corners can take place due to ion bombardment [38,43]. Since the sputtering yield is a function of the ion incident angle, the sharp corners may erode, causing the mask to effectively have some tapering along its sidewalls [44]. This effect can be reduced by having thick masks, but this is not always feasible, especially since the mask thickness also has an effect on the resulting profile of the etched feature.

**Figure 9:**
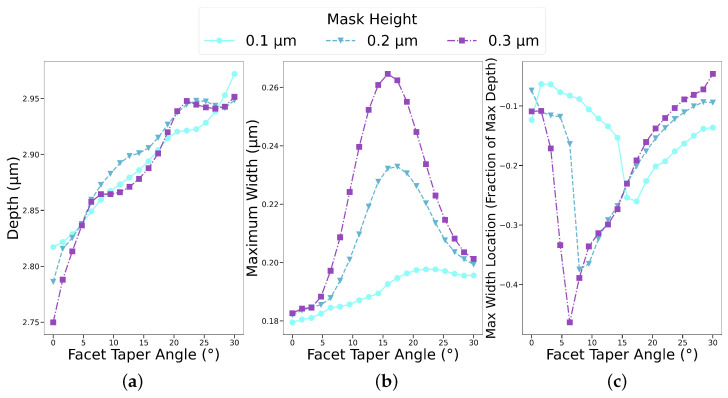
Depth, maximum width, and its location down the feature sidewall for thin mask faceting. In (**a**), the maximum depth increases with increasing the tapering angle. In (**b**), bowing is at its maximum for angles between 15° and 20° while being less pronounced for the thinnest considered mask of 0.1 μm due to a reduced ion reflection surface. The location of the maximum width (**c**) jumps from just under the mask towards the bottom of the profile and approaches the mask again for higher angles.

We considered thin masks, where ion sputtering fully eroded the top corner, leading to a tapering along the entire thin mask sidewall, as shown in Figure 5a. Due to the high selectivity of silicon etching versus mask etching, which is well above 100 [45,46], masks thinner than 0.1 μm may be employed in a SF_6_/O_2_ plasma; nevertheless, we used 0.1 μm as the minimum examined thickness, which still caused a significant sidewall etching, and the thinner masks’ impacts did not differ significantly in their anisotropic nature. In Figure 9, we observe the effective changes induced in the etched profile due to faceting of a thin mask in a plasma with an oxygen fraction y_O_2__ = 0.5 in the feed gas by capturing the maximum depth and overall profile width, as well as the normalized location along the profile where the maximum width occurred. The value of 0 in the depth axis of Figure 9c represents the bottom of the mask and −1 is the bottom of the final geometry. The tapering angle was varied from 0° to 30° to capture the impact of the mask’s top-corner sputtering at its extremes. Higher faceting angles resulted in a fully directional vertical etching as no ion were reflected from the mask towards the substrate sidewall, resulting in the same profile as without faceting. In Figure 9, we observe that the maximum depth increased when increasing the facet taper angle. The increase was not completely linear as it depended on the location where the first and second ion reflections off the sidewall landed.

Subsequently, the bowing effect, shown in Figure 9b, reached its maximum value between 15° and 20° while being less pronounced for the thinnest considered mask of 0.1 μm, since there was a smaller mask sidewall area from which the ions could be reflected. Furthermore, we noted the location down the feature sidewall where the maximum width occurred in Figure 9c. At very small angles, which corresponded to weak faceting, the maximum width occurred just under the mask as the reflected ions impinged directly at the bottom of the geometry, leaving the top of the substrate most exposed to lateral etching. A further increase in tapering shifted the impact point of the reflected ions from the feature bottom towards the sidewall, resulting in a steep decrease in the vertical location of the maximum width. Analogous to the previous section, very high angles shifted the bowing effect further up the profile sidewall. This showed that the sputtering of the mask corners could lead to significant changes in the final profile. By using significantly thicker masks, a smaller fraction of the mask would be faceted and the impact would be less pronounced.

### 4.3. Impact of Mask Etch Rate

**Figure 10:**
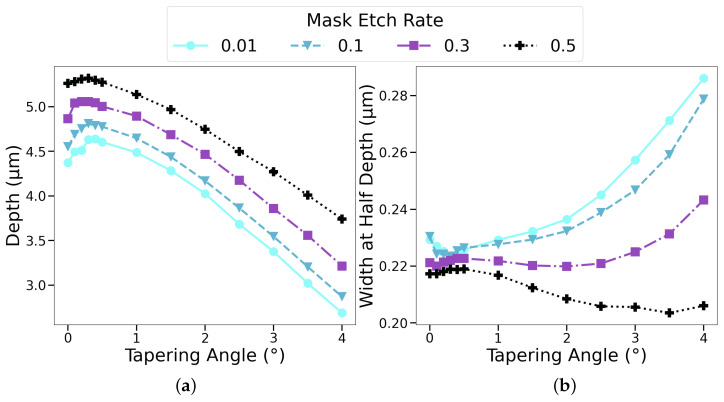
Impact of the mask etch rate on the depth and WAHD of the etched profile. (**a**) The highest vertical etch rate is achieved when the entire mask is etched away. (**b**) Consequently, this scenario results in the lowest lateral etch rate.

In Section 4.1, the applied mask sputtering yield used was calibrated to the experiments with the SiO_2_ mask. Since different mask materials have different sputtering yields, using the same setup from Section 3 with oxygen fraction y_O_2__ = 0.5 in the feed gas, we varied the total mask sputtering yield from 0.01 to 0.5, corresponding to negligible mask etching and etching away the entire mask layer, respectively. In Figure 10a, we observe that the longest depth was achieved in the scenario where the entire mask was etched away (sputtering yield of 0.5), while resulting in the lowest lateral etching, as shown in Figure 10b. This was in line with the findings from the previous sections, as thinner masks caused less species reflections towards the sidewall.

### 4.4. Rectangular Trench Profiles

The results presented in the previous sections focused on cylindrical hole geometries. We now turn our attention to the trench masks, with a trench width of 0.4 μm using the parameters from Table 1.

**Figure 11:**
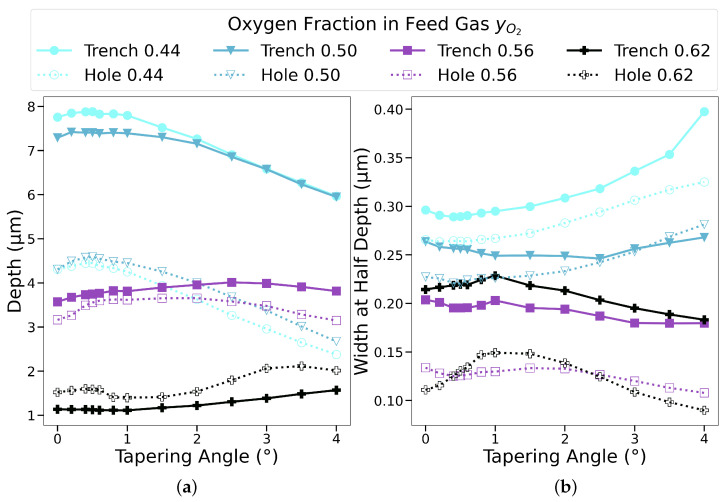
(**a**) Depth and (**b**) width at half-depth (WAHD) for trenches denoted with a solid line with squares, and holes shown with a dashed line with circles. Trench geometries exhibit higher vertical and lateral etching.

From the results in Figure 11a, we can observe that the trenches had significantly higher vertical etch rates than their comparable hole geometries, while at the same time having higher lateral etch rates in the vast majority of the samples. Higher etch rates in both metrics could be explained by a higher flux of all species reaching the substrate surfaces in trenches.

The reason for the increased flux in the trench geometry is due to less reflections taking place off the sidewalls in a trench geometry. When a particle enters a trench, its movement is only restricted in one dimension, while a cylindrical geometry interrupts a particle’s motion in all directions. This means that a particle which finally causes the removal of a substrate will have undergone significantly more reflections off of the sidewall, meaning that its effective weight will be reduced. In the case where the oxygen fraction is very high (0.62), the effect is less significant or even reversed. This is because the increased oxygen inhibits chemical reactions and almost all of the etching is then caused by ion bombardment. As ions hit the surface of the sidewall, they are directed towards the bottom, resulting in a deeper profile in the cylindrical geometry due to more surfaces being present, off of which the ions can reflect specularly and be directed towards the trench bottom.

Regarding the impact of the mask taper angle, we note that the general trend is the same for both cylindrical and trench geometries. Increasing the taper angle beyond 1° results in a shallower geometryx, when using either a cylindrical or rectangular trench mask. Therefore, the results presented earlier for a cylindrical hole are also valid for a trench structure.

## 5. Conclusions

A thorough simulation study of the impact of the mask geometry on the plasma etched profiles for cylindrical holes and rectangular trenches was performed. This required the implementation of a physics-based 3D feature-scale model for SF_6_/O_2_ plasma etching in an in-house level set-based process simulator, ViennaPS, and its calibration using experimental data from literature. The model was subsequently applied to study the impact of mask tapering, mask thickness, and faceting on the final feature profiles.

It is well established that the increase of O_2_ in the feed gas makes the etching process more anisotropic. We found that slight variations in the taper angle, up to 1° for both cylindrical holes and rectangular trenches, either increased the directionality of the etching process or did not impact the final profile significantly. Moreover, having a taper angle of 0.5° was even desirable as it led to more directional etching and deeper etched profiles. Therefore, for the masks with about 1.2 μm in thickness, mask design rules can be relaxed as long as the angle does not exceed 2°, at which point significant lateral etching can lead to undesirable bowing in the final structure. The effect of the variations in the taper angle can be mitigated by introducing more passivating species into the feed gas; however, this will lead to a significant increase in the etch time since the passivating species serves to inhibit etching everywhere in the trench.

In the mask faceting study, we used the same approach to examine the fabrication parameters with different mask materials, which caused changes in the ion sputtering of the mask, leading to faceting. The impact of the very high tapering angles on thin masks with thicknesses in the range 0.1 μm to 0.3 μm were simulated. We found that allowing a variation in the design of the mask sidewall tapering, up to 10°, did not significantly impact the etching profile, meaning that the mask design rules could be relaxed there. However, one must ensure that sidewall tapering does not exceed 10°, as this will cause significant bowing in the final structure. Additionally, it is beneficial to use as thin masks as possible, 0.1 μm and below, as they naturally have less of an impact on the final structure. The impact of the ion sputtering can be attenuated by using very thick masks as the erosion of the top corners will not have such a pronounced effect. However, this is not feasible for all materials and devices.

Finally, to have a closer look at the impact of the mask etch rate, we compared several scenarios between negligible mask etching and complete mask removal by the end of the process. Analogous to the finding that thin masks had less of an impact, we found that masks which were easily etched away produced less lateral etching. Hence, mask erosion does not necessarily lead to undesired effects, but it is essential to ensure it does not get completely removed during the etching process, as this would lead to substrate etching in undesired places.

## Figures and Tables

**Table 1:** Parameters used for the mask study, which correspond to a chamber with a total gas flow rate of 80 sccm at a pressure of 25 mTorr, an inductive coil power of 800 W, and an RF-bias voltage of −120 V at a wafer temperature of 5 °C. The gas flow rates of the individual gases were 40 sccm SF_6_ and 40 sccm O_2_, as described in [25].

Parameter	Oxygen Fraction in Feed Gas y_O_2__				Unit
	0.44	0.5	0.56	0.62	
Fluorine flux $\Gamma_{F}$	5.5 × 10^18^	5 × 10^18^	4 × 10^18^	3 × 10^18^	cm^−2^s^−1^
Oxygen flux $\Gamma_{O}$	2 × 10^17^	3 × 10^17^	1 × 10^18^	1.5 × 10^18^	cm^−2^s^−1^
Ion flux $\Gamma_{i}$	1 × 10^16^	1 × 10^16^	1 × 10^16^	1 × 10^16^	cm^−2^s^−1^
F sticking on Si $\gamma_{F}$	0.7	0.7	0.7	0.7	1
O sticking on Si $\gamma_{O}$	1.0	1.0	1.0	1.0	1
Si density $\rho_{Si}$	5 × 10^22^	5 × 10^22^	5 × 10^22^	5 × 10^22^	atoms cm^−3^
O recombination rate $\beta \sigma_{Si}$	5 × 10^13^	5 × 10^13^	5 × 10^13^	5 × 10^13^	cm^−2^s^−1^
Reaction rate constant $k\sigma_{Si}$	3 × 10^17^	3 × 10^17^	3 × 10^17^	3 × 10^17^	cm^−2^s^−1^
Si yield proportionality constant $A_{Si}$	7.0	7.0	7.0	7.0	1
O yield proportionality constant $A_{O}$	2.0	2.0	2.0	2.0	1

## Data Availability

The data presented in this study is available upon request from the corresponding author. The simulation tool used in the study, ViennaPS, is available via open source license: https://github.com/ViennaTools/ViennaPS (accessed on 2 January 2023).

## Abbreviations

The following abbreviations are used in this manuscript:

3D	three-dimensional
AM	additive manufacturing
DRIE	deep reactive ion etching
DTCO	design-technology co-optimization
ER	etch rate
FET	field-effect transistor
HAR	high aspect ratio
RF	radio frequency
RIE	reactive ion etching
SDF	signed distance function
SEM	scanning electron microscopy
TSV	through-silicon via
WAHD	width at half-depth
